# Genome maintenance and transcription integrity in aging and disease

**DOI:** 10.3389/fgene.2013.00019

**Published:** 2013-02-25

**Authors:** Stefanie Wolters, Björn Schumacher

**Affiliations:** ^1^Cologne Excellence Cluster for Cellular Stress Responses in Aging-Associated Diseases, Institute for Genetics, University of CologneCologne, Germany; ^2^Systems Biology of Ageing Cologne, University of CologneCologne, Germany

**Keywords:** DNA repair, progeria, transcription, genetic, DNA damage, cancer

## Abstract

DNA damage contributes to cancer development and aging. Congenital syndromes that affect DNA repair processes are characterized by cancer susceptibility, developmental defects, and accelerated aging (Schumacher et al., 2008). DNA damage interferes with DNA metabolism by blocking replication and transcription. DNA polymerase blockage leads to replication arrest and can gives rise to genome instability. Transcription, on the other hand, is an essential process for utilizing the information encoded in the genome. DNA damage that interferes with transcription can lead to apoptosis and cellular senescence. Both processes are powerful tumor suppressors (Bartek and Lukas, 2007). Cellular response mechanisms to stalled RNA polymerase II complexes have only recently started to be uncovered. Transcription-coupled DNA damage responses might thus play important roles for the adjustments to DNA damage accumulation in the aging organism (Garinis et al., 2009). Here we review human disorders that are caused by defects in genome stability to explore the role of DNA damage in aging and disease. We discuss how the nucleotide excision repair system functions at the interface of transcription and repair and conclude with concepts how therapeutic targeting of transcription might be utilized in the treatment of cancer.

## GENOME MAINTENANCE DEFECTS CAUSE CANCER SUSCEPTIBILITY AND PREMATURE AGING

**FIGURE 1:**
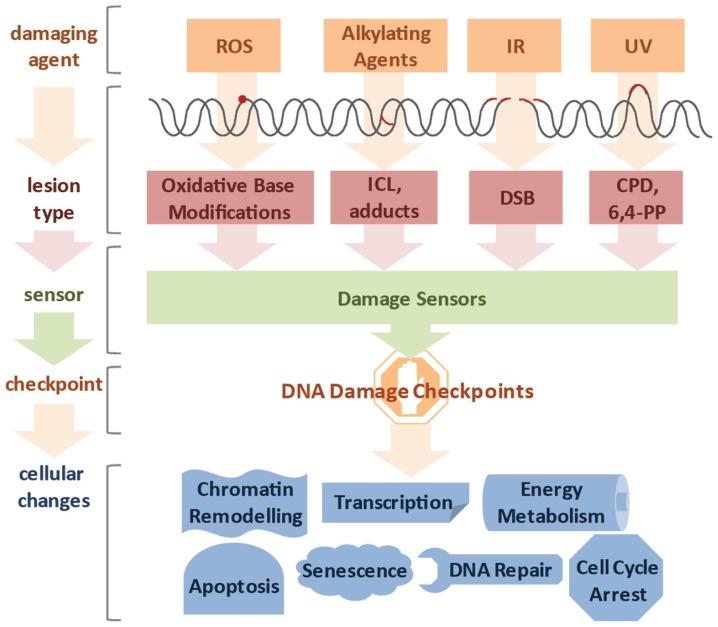
**Diverse lesion types trigger DNA damage responses**. DNA damage can be caused by various genotoxic agents, such as reactive oxygen species (ROS) produced during cellular metabolism, alkylating agents that find application in cancer therapy, ionizing irradiation (IR), which is used for radio therapy, or ultraviolet (UV) irradiation presenting a daily threat as it is contained in sunlight. The inflicted lesions are just as diverse, since ROS usually lead to base modifications; alkylating agents form adducts, while bifunctional alkylating agents crosslink DNA to form interstrand crosslinks (ICLs). IR typically induces double-strand breaks (DSBs), and UV light triggers the formation of cyclobutane pyrimidine dimers (CPDs) and 6,4-photoproducts (6,4-PPs). Cells have a repertoire to sense the different lesions and subsequently activate DNA damage checkpoint proteins. Ultimately, cells respond to the DNA damage by chromatin remodeling, modified transcription, fine-tuning of energy metabolism, cell cycle arrest, activation of DNA repair pathways and in case of irreparable damage load, induction of senescence or apoptosis.

It was estimated that DNA damage occurs on the order of tens of thousands per genome on a daily basis (Lindahl and Nyberg, 1972). Genotoxic insults can stem from a large variety of endogenous and exogenous sources (**Figure 1**). Cellular metabolism can produce reactive oxygen species (ROS) and alkylating agents, while cells can be exposed to ultraviolet (UV), ionizing radiation (IR), and a variety of genotoxic chemicals (Loeb and Harris, 2008). The type of lesion can vary widely and depends on the source of DNA damage. For example, ROS induces oxidative base modifications, IR typically leads to single- and double-strand breaks (SSB and DSB, respectively), DNA alkylation can lead to adduct and interstrand crosslink (ICL) formation, and UV triggers the formation of thymidine dimers (Hurley, 2002). The toxicity of DNA damage depends on the structural changes they inflict as well as the characteristics of the cell they occur in. Proliferating cells have a different repertoire of DNA repair pathways than quiescent cells and, therefore, the same lesion might have different effects in different tissues. In cycling cells, for instance, a single DSB is sufficient to impair chromosome segregation during mitosis and ICLs lead to replication fork collapse. For these reasons even a small number of DSBs and ICLs can be cytotoxic. In contrast, oxidative base modifications are generally less obstructive, while UV-induced cyclobutane pyrimidine dimers (CPDs) can be read through by specialized DNA polymerases and thus, can persist through replication, but pose an obstacle to transcription and lead to stalling of RNA polymerases (RNAP).

Given the frequency and impact of DNA damage, highly sophisticated DNA repair systems have evolved. These systems recognize specific types of lesions and induce DNA damage signaling. Failure of DNA repair has been associated with severe disorders in humans, often associated with occurrence of cancer and/or premature aging (**Table 1**).

**Table 1:** Human disorder associated with genome maintenance defects.

Disorder	Abbr.	Gene(s) mutated	Pathway impaired	Enhanced cancer-susceptibility	Progeroid features
Ataxia telangiectasia	AT	ATM	DSB repair	+	+
Ataxia telangiectasia-like disorder	ATLD	MRE11	DSB repair	-	(+)
Atypical Werner syndrome	WS	WRN	nuclear structure	+	+
Bloom’s syndrome	BLS	BLM	recombination Q (RECQ) helicase	+	+
Cerebro-oculo-facio-skeletal syndrome	COFS	CSB, xeroderma pigmentosum D (XPD), XPG, ERCC1	TC-NER	-	+
Cockayne syndrome	CS	CSA, CSB	TC-NER	-	+
Cockayne syndrome type II	CS type II	CSA, CSB	TC-NER	-	+
Dyskeratosis congenita	DKC	DKC1, TERC1	Telomere maintenance	+	+
Fanconi anemia	FA	FANCA, B, C, D1 (*BRCA2)*, D2, E, F, G, I, J (*BRIP1*), L, M, N (*PALB2)*, O (*RAD51C)*, and P (*SLX4)*	ICL repair	+	+
Hutchinson–Gilford-progeria/syndrome	HGPS	LMNA, ZMPSTE24	nuclear structure	-	+
Li–Fraumeni Syndrome		p53	many (p53 tumor suppressor inactivation)	+	-
Mandibuloacral dysplasia	MAD	LMNA, ZMPSTE24	nuclear structure	-	(+)
Nijmegen breakage syndrome	NBS	NBS1	DSB repair	+	(+)
Restrictive dermopathy	RD	ZMPSTE24	nuclear structure	-	+
Rothmund–Thomson syndrome	RTS	RECQL4	RECQ helicase	+	+
Trichothiodystrophy	TTD	TFIIH complex: XPD, xeroderma pigmentosum B (XPB), GTF2H5 trichothiodystrophy A (TTDA), MPLKIP (PLK1)	NER	-	+
UV-sensitive syndrome	UVSS	UVSSA	NER	-	(+)
Werner syndrome	WS	WRN	RECQ helicase, influencing nuclear structure, DSB repair, ICL repair, MMR, BER, TLS	+	+
Xeroderma pigmentosum	XP	XPA-G	NER	+	-

### DSB REPAIR AND THE DNA DAMAGE RESPONSE

The DNA damage response (DDR) to DSBs is particularly well-understood. As the presence of a DSB poses a major obstacle for further cell division a sophisticated network of DDR signaling is ignited (Ciccia and Elledge, 2010). Genetic experiments that were performed in yeast nearly 25 years ago established that DNA damage checkpoints transiently halt cell cycle progression in the presence of genotoxic stress to assure that the repair is completed before cell division (Weinert and Hartwell, 1989; Rowley et al., 1992). The recognition that DDR defects are causal for cancer development has sparked major research efforts employing model systems from yeast to mammals. The DNA damage checkpoint mechanisms turned out to be highly conserved throughout evolution. Multicellular organisms, however, not only respond by transient cell cycle arrest but also by inducing cellular senescence, thus permanently withdrawing from cell division, and apoptosis, through which damaged cells commit suicide to no longer pose a threat to the organism (Harper and Elledge, 2007). Intriguingly, the DDR not only impacts on regulators of cellular proliferation and cell death but impinges on a variety of cellular processes such as transcription, DNA repair, respiration, energy metabolism, chromatin remodeling, and others (**Figure 1**; Jackson and Bartek, 2009).

The initial recognition of DSBs involves binding of the trimeric Mre11–Rad50–Nbs1 (MRN) complex consisting of MRE11, NBS1, and RAD50 to the broken DNA ends (Bartek and Lukas, 2007). The MRN complex activates the PI3 kinase-like kinase ataxia telangiectasia mutated (ATM), which in turn phosphorylates a plethora of targets (Shiloh, 2003). ATM targets include the checkpoint kinase CHK2, which in turn activates p53. p53 then induces cell cycle arrest and amid severe damage apoptosis. Mutations in p53 underlie Li–Fraumeni syndrome that causes a strong cancer predisposition (Vogelstein et al., 2000). About half of human cancers carry acquired mutations in p53, making it a key tumor suppressor. As the importance of p53 in cancer development has been recognized, research activity has been continuously expanding on this tumor suppressor molecule. Indeed, p53 has been implicated in the regulation of a number of cellular DDR outputs and more recently also in regulating non-cell autonomous responses to DNA damage (Reinhardt and Schumacher, 2012).

Cells with defective DSB repair, like ATM mutants, are highly IR sensitive. Heritable mutations in ATM result in ataxia telangiectasia (AT), a rare recessive syndrome that is associated with progressive neurodegeneration, variable kinds of immune-deficiencies and a predisposition to lymphoid cancer (Lavin, 2008). Patients with mutated NBS1 develop Nijmegen breakage syndrome (NBS) and exhibit similar symptoms as seen in AT. Most frequently the binding site of NBS1 to MRE11 and therefore the MRN complex formation is disrupted (Maser et al., 2001). In mice, complete loss of NBS1 is embryonic lethal, which is consistent with embryonic lethality of MRE11 and RAD50 null mutants (Xiao and Weaver, 1997; Friedberg and Meira, 2006). Nevertheless, mutations affecting, but not abolishing, the binding of MRE11 to the other two subunits of the MRN complex lead to ataxia telangiectasia-like disorder (ATLD) showing phenotypes distinct from NBS. This suggests that MRE11 and NBS1 might have functions independent from the MRN complex (Taylor et al., 2004).

While DNA damage checkpoint signaling halt the cell cycle, at least two distinct DSB repair machineries are activated depending on the phase of the cell cycle (Chapman et al., 2012). In S/G2 phase homologous recombination (HR) uses the sister chromatid as template for accurate repair, while during G1 non-homologous end joining (NHEJ) ligates the broken ends after end resection. Thereby NHEJ comprises a fast and efficient but error prone DNA repair method. However, for large parts of the genome the NHEJ-induced errors can be tolerated when genes are not affected. Since NHEJ is utilized when no sister chromatid is available, it is thought to function mainly in non-proliferating cells like neurons (Jeppesen et al., 2011). Null mutations of NHEJ pathway proteins XRCC4 and LIG4 are embryonic lethal in mice. In 2001, LIG4 syndrome has been described in humans that shares phenotypic similarities with NBS but lacks cancer predisposition (O’Driscoll et al., 2001).

In the case of HR, both, reduced and elevated activity, have been associated with a predisposition to cancer (Modesti and Kanaar, 2001). For example, dysfunctional BRCA1 and BRCA2 diminish the efficiency of HR and germ line mutations in humans lead to a high incidence of breast cancer (Moynahan et al., 1999, 2001). Mouse models for Bloom’s syndrome (BLS), in contrast, display hyperactive HR and exhibit a high tumor susceptibility (Luo et al., 2000). It thus became apparent that the fine-tuning of HR is essential for maintaining genome stability.

### ICL REPAIR

Like DSB repair, the employment of removal mechanisms of ICLs alters depending on cell cycle stage. During G1 the excision repair cross-complementation group 1–xeroderma pigmentosum group F (ERCC1–XPF) endonuclease initiates the ICL removal (Deans and West, 2011). When ICLs are encountered by the replication fork a Fanconi anemia (FA) protein complex comprised of FANCA, -B, -C, -E, -F, -G, -L, and -M mono-ubiquitylates FANCD2 that interacts with DSB repair proteins including the breast cancer susceptibility gene BRCA1, FANCD1/BRCA2, FANCJ, and the MRN complex (Kee and D’Andrea, 2010). BRCA1/FANCD2 and RAD51/FANCD2 complexes accumulate at the site of damage and form foci during S-phase of cell cycle. Subsequent repair is thought to be achieved by HR pathway (Taniguchi et al., 2002).

The inability to repair ICLs in FA patients leads to replication fork collapses, particularly in actively dividing cells such as the hematopoietic system. Consequently, FA is characterized by bone marrow failure due to chromosomal aberration and leukemic transformation of cells. Also FA is characterized by cancer predisposition and hypersensitivity to crosslinking agents like mitomycin C, cisplatin, or diepoxybutane (Wang and Gautier, 2010).

### RecQ HELICASES AND DNA REPAIR

Also the Werner protein (WRN) has been linked to DNA repair when ICLs cause replication fork breakdown (Otterlei et al., 2006). WRN has a recombination Q (RecQ) helicase and an exonuclease domain and functions during replication and recombination repair. Indeed, WRN has been shown to interact with proteins of many different DNA repair pathways such as HR via RAD52 (Baynton et al., 2003), BRCA1 (Cheng et al., 2006), RAD51 (Otterlei et al., 2006), and NSB1 (Cheng et al., 2004), NHEJ via Ku70–80 (Cooper et al., 2000) and LIG4–XRCC4–XLF (Kusumoto et al., 2008) complex, base excision repair (BER) via PARP1 (von Kobbe et al., 2003) and mismatch repair (MMR) via interaction with MSH2/MSH6, MSH2/MSH3, and MLH1/PMS2 complexes (Saydam et al., 2007). Taken together, this indicates that WRN might be a multifunctional protein required during the repair of a variety of DNA lesions.

Mutations in *WRN* underlie Werner syndrome (WS), which is a rare autosomal recessive disease. WS patients not only exhibit elevated cancer predisposition, but also develop symptoms of premature aging. The progeroid features of WS patients are particularly well recognized as a premature onset of aging as they develop typically during the third decade of life. Classic forms of progeroid syndromes are also found in patients carrying mutations in related RecQ helicases. BLS, and Rothmund–Thomson syndrome (RTS) are caused by mutations in the Bloom’s, and RECQL4 helicases, respectively (Hickson, 2003; Bohr, 2008). Similar to WS, BLS and RTS patients show a wide variety of progeroid features as well as elevated cancer susceptibility.

### TELOMERE MAINTENANCE

Genome instability can also be caused by shortening of telomeres. Telomeres are the end-capping structures that maintain the integrity of linear chromosomes. With each cell cycle the telomeres become shorter due to the end replication problem and require telomerase for maintenance (de Lange, 2009). Most somatic cells do not express telomerase and, consequently, progressive shortening limits the replicative lifespan of somatic cells. Critically shortened telomeres are recognized as DSB and induce checkpoint signaling leading to cellular senescence (Abdallah et al., 2009). Cancer cells often re-express telomerase allowing them to continuously grow. Also stem cells and germline compartments express telomerase to maintain their replicative potential. Mutations in the telomerase complex components, DKC1 and TERC1, lead to dyskeratosis congenita (DKC), hallmarks of which include growth and mental retardation, immune deficiency, and anemia (Marrone et al., 2005; Armanios and Blackburn, 2012). Telomere length appears to be correlated with life expectancy (Cawthon et al., 2003), fueling the proposition that telomere length could serve as predictive marker of biological aging.

### NUCLEAR INSTABILITY AS A SOURCE FOR GENOTOXIC STRESS

Genome instability can also result from mechanical stress on the nucleus. So called laminopathies such as Hutchinson–Gilford-progeria-syndrome (HGPS), atypical WS, restrictive dermopathy (RD), and mandibuloacral dysplasia (MAD) are caused by mutations in lamin A and the ZMPSTE24 farnesyltransferase required for lamin A processing (Ramirez et al., 2007). The nuclear instability leads to DNA damage accumulation and evokes a DDR. HGPS fibroblasts as well as mouse embryonic fibroblasts (MEFs) from *Zmpste24*^-/-^ mice show elevated chromosomal instability and DNA damage sensitivity (Liu et al., 2005). It was suggested that the nuclear envelope instability in HGPS interferes with the correct localization of the MRN complex and the recruitment of the DSB repair factors 53BP1 and RAD51 (Liu et al., 2005; Constantinescu et al., 2010). Consistent with enhanced genome instability, mutations in p53 can partially alleviate the HGPS pathology in mice (Varela et al., 2005). In addition to interfering with DNA repair, nuclear envelope instability might also impair other essential nuclear processes such as chromatin localization and modifications and thus, gene expression (Burgess et al., 2012). Together this combination might be accountable for the severe and highly complex disease manifestation that characterizes laminopathies.

### NUCLEOTIDE EXCISION REPAIR

Mutations in nucleotide excision repair (NER) underlie a variety of skin cancer predisposing and degenerative disorders (Cleaver et al., 2009; **Table 1**). Unlike most of the progeroid syndromes discussed above, mutations that affect the two distinct branches of NER are linked either to cancer susceptibility or to premature aging. Defects in the global genome (GG-) NER branch cause the skin cancer susceptibility syndrome xeroderma pigmentosum (XP), while mutations affecting the transcription-coupled (TC-) NER branch lead to progeroid syndromes such as Cockayne syndrome (CS) that is characterized by postnatal growth retardation and accelerated aging but not cancer (Lehmann, 2003). The distinct TC-NER pathologies suggest that transcriptional impediments might be particularly relevant to the aging process.

## TRANSCRIPTION-COUPLED REPAIR IN AGING AND DISEASE

**FIGURE 2:**
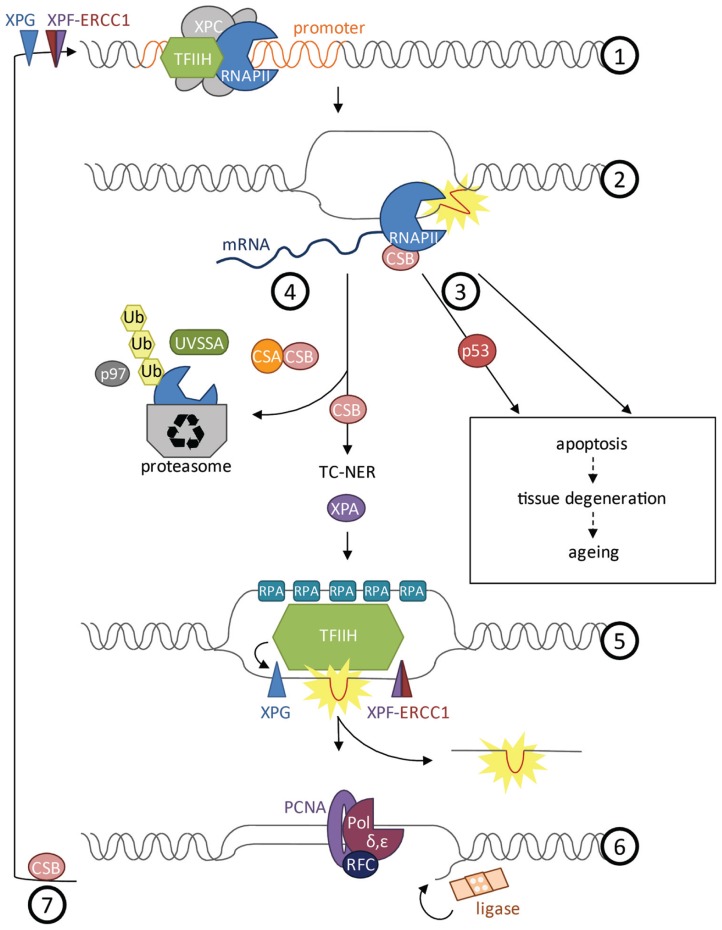
**Transcription at damaged DNA**. (1) Prior to formation of the transcription initiation complex, XPF–ERCC1 and XPG cut the DNA proximal to the promoter to get ready for transcription initiation. TFIIH unwinds the DNA to open the promoter during initiation phase. (2) CSB is bound to and moves with RNAPII during elongation phase and can activate repair when the polymerase gets stalled at a lesion. (3) Depending on the type of damage, cells will undergo apoptosis upon stalling of RNAPII. Apoptosis can be induced in a p53-dependent or -independent manner and might lead to tissue degeneration and aging of the organism. (4) Opposing induction of cell death, CSB can induce repair of the lesion by TC-NER. Removal of RNAPII is a prerequisite for repair that is dependent on CSB and CSA. CSA is recruited by CSB. UVSSA as well as NEDD4/Rsp5 and Cul3 are important for polyubiquitylation of RNAPII via Cdc48/p97 and for stabilization of CSB at the site of damage. RNAPII is degraded by the proteasome while CSB recruits the factors of the downstream NER pathway. (5) XPA validates the lesion and TFIIH unwinds the helix around the lesion. Replication protein A (RPA) coats the single-stranded DNA to prevent the strand from reacting with other factors or forming secondary structures. XPG is recruited 3’ of the damaged DNA and stabilizes the XPF–ERCC1 endonuclease complex 5’ of the lesion. A fragment of 25–30 nucleotides length containing the lesion is cut and released. (6) Finally the gap is filled by DNA polymerase δ and ε, recruited by replication factor C (RFC) and proliferative cell nuclear antigen A (PCNA), and the nick is sealed by DNA ligase. (7) Re-initiation of transcription upon UV irradiation requires CSB.

When the RNAPII is released from the transcription initiation complex into the elongation phase it does not move uninterrupted along the coding sequence (Larson et al., 2011). *In vivo* experiments monitoring transcription elongation speed have revealed that RNAPII often stalls even in the absence of exogenous DNA damage. Transcription appears rather like stop-and-go traffic than a continuous process. This might have to do with spontaneous DNA damage, structural tension, histone remodeling, or regulatory events, all of which might impede elongation. During elongation, the transcription complex might also serve as control mechanism for DNA integrity, particularly in post-replicative cell types. The sensitivity of ongoing transcription to stall at DNA adducts was suggested to function as a “damage dosimeter” (Ljungman and Lane, 2004). When RNAPII stalls at a lesion, TC-NER initiates the NER reaction to remove a stretch of the damaged strand (Hoeijmakers, 2001; **Figure 2**). The CSB protein, comprising a switch/sucrose nonfermentable (SWI/SNF)-like DNA-dependent ATPase, is associated with RNAPII and upon stalling recruits the WD40 domain protein CSA. CSB is stabilized by the recently discovered UV-stimulated scaffold protein A (UVSSA) protein that ubiquitylates the stalled RNAPII (Nakazawa et al., 2012; Schwertman et al., 2012; Zhang et al., 2012). The CSB-dependent TC-NER complex recruits the NER machinery including XPA and the 10-subunit transcription factor II H (TFIIH) complex that comprises XPB, XPD, and TTDA (p8). TFIIH locally unwinds the DNA and recruits XPG to the 3′ side of the lesion, which in turn stabilizes binding of the XPF–ERCC1 heterodimer (also called XFE) 5′ to the lesion. Both XFE and XPG are endonucleases that incise the damaged strand 25–30 nucleotides apart. The single-stranded stretch is coated by RPA before the gap is filled by DNA polymerases δ and ε that are recruited through RFC and PCNA. Finally, the nick is sealed by DNA ligase. In response to transcription stalling also p53 can be activated to arrest the cell cycle or induce apoptosis (Ljungman, 2000). As part of resolving the transcriptional impasse, degradation of the stalled RNAPII is induced by NEDD4/Rsp5 and Cul3-dependent ubiquitylation and subsequent proteasome targeting through Cdc48/p97 (Anindya et al., 2007; Verma et al., 2011). Outside of actively transcribed genes the XPC protein scans for UV-induced lesions. Upon lesion detection the XPC–RAD23 and the DDB1–DDB2 complex recruit the same NER machinery as the TC-NER complex (Cleaver et al., 2009).

Intriguingly, even though any mutation in NER genes confers cellular UV sensitivity, the pathological consequences of mutations affecting TC-NER or GG-NER are fundamentally different (Hanawalt and Spivak, 2008). Most mutations in the TC-NER factors CSA and CSB lead to CS that is characterized by developmental defects and premature aging with an onset at 3–4 years of age. These classic types of CS exhibit neurological abnormalities, limb ataxia, inner ear defects, cachexia, retinal degeneration, postnatal growth retardation, progressive kyphosis, ataxia, and photosensitivity but do not develop skin cancer (Laugel et al., 2010). Mutations in CSA and CSB can also cause the more severe CS type II as well as Cerebro-oculo-facio-skeletal syndrome (COFS) that is diagnosed at birth with craniofacial and skeletal abnormalities, severely reduced muscle tone, and impairment of reflexes. A third disorder caused by mutations in CSA, CSB, and also UVSSA is UV-sensitive syndrome (UVSS). UVSS patients show photosensitivity and mild freckling but no skin cancer predisposition or neurological abnormalities. So far it has remained elusive how nature and location of different mutations in CSA or CSB are linked to the diverse phenotypic outcomes of the disease. Certainly, stalled RNAPII complexes elicit a strong pro-apoptotic signal. However, cell loss through apoptosis only explains a limited part of the complex human diseases caused by TC-NER defects. Consistent with a strong pro-apoptotic signal emanating from stalled RNAPII, *Csb *mutations reduced cancer development in a tumor prone mouse model (Lu et al., 2001). Mouse models for the CS-related trichothiodystrophy (TTD) were protected from tumor development (Wijnhoven et al., 2007). Taken together, these observations suggest that transcription impediments can have tumor suppressive consequences.

In contrast to CS, a variety of mutations in the NER pathway can give rise to XP that is characterized by pigmentation abnormalities, photosensitivity, skin atrophy, and a 1000-fold elevated skin cancer susceptibility (DiGiovanna and Kraemer, 2012). So far mutations in seven XP genes, XPA-G, were found in XP patients. Intriguingly, mutations in XPC give rise to the specific skin phenotypes in XP patients, while mutations in XP genes that function in the NER pathway downstream of GG-NER and TC-NER, can result in additional neurodegenerative phenotypes. The mutation spectrum of the XPD gene is particularly striking. Mutations in the XPD subunit of TFIIH can give rise to XP, XP combined with CS (XPCS), and TTD. The TTD pathology that results from mutations in *XPB *and *XPD* includes neurological and skeletal degeneration, cachexia, ichthyosis (scaling of the skin), and characteristic brittle hair and nails. Especially the brittle structure of the hair and nails has been linked to defects in completing transcription leading to deficiency of high-sulfur matrix proteins (Hoeijmakers, 2001; Lehmann, 2003).

Notably, neurodegeneration is a very common trait not only of NER deficiencies but it is associated with many of the disorders that are caused by DNA repair defects. It is conceivable that neurons are highly susceptible to DNA damage. Most neurons are irreplaceable and thus, permanently lost when removed by apoptosis. Moreover neurons exhibit a high oxygen metabolism rate and low antioxidant levels (Barzilai et al., 2008), which might elicit genotoxic stress in this cell type.

## TISSUE MAINTENANCE AT THE COST OF RENEWAL AND GROWTH

DNA damage checkpoint mediated proliferation arrest and apoptosis can explain some of the degenerative features of genome instability syndromes. However, the wide variety of phenotypic manifestations in the diverse progeroid syndromes suggests that more complex organismal responses are involved. Indeed, recent studies in mice have revealed endocrine adjustments that might explain some of the disease outcomes. Mouse models for CS and XFE progeria as well as HGPS mice and mutants in *Sirt6 *exhibit severe growth defects and degenerative phenotypes mirroring the human disorders (Mostoslavsky et al., 2006; Niedernhofer et al., 2006; van der Pluijm et al., 2006; Mariño et al., 2010). The histone deacetylase Sirt6 is required for genome stability, likely through its role in DSB repair (Kaidi et al., 2010). Intriguingly, all those mouse mutants show low circulating levels of the insulin-like growth factor (IGF)-1. IGF-1 is an essential component of the somatotropic axis that regulates body growth through IGF-1 receptor (IGF-1R) mediated mitogenic signaling (Carter et al., 2002). IGF-1 secretion is triggered by growth hormone receptor (GHR) activation in response to pituitary GH. Abrogated GHR signaling either due to pituitary defects leading to failure of GH production or knockout of *Ghr* itself leads to severely reduced body growth but extension of lifespan (Bartke, 2009). In fact mutations in *Igf-1r* confer lifespan extension in worms, flies, and mammals indicative of a highly conserved longevity assurance pathway (Kenyon, 2001). Mechanistically, it was demonstrated that in response to transcription-blocking lesions cells downregulate GHR and IGF-1R levels and activity (Garinis et al., 2009). This response conferred reduced cell proliferation and enhanced stress resistance. Persistence of transcription-blocking lesions even at low levels leads to a prolonged GHR/IGF-1R attenuation. The reduction of somatotropic signaling might shift the endocrine environment from growth to maintenance, thus accounting for growth failures and perhaps also contributing to the tumor protection, for instance in CS patients (Schumacher et al., 2008; Schumacher, 2009). Enhanced cellular stress resistance on the other hand, is associated with extended longevity in various species including mice. It was thus proposed that amid persistent DNA damage organisms evoke a “survival” response through somatotropic attenuation to preserve tissue maintenance and antagonize the detrimental consequences of genome instability (Schumacher et al., 2008).

## AT THE INTERFACE OF TRANSCRIPTION AND REPAIR

Mechanistically, the complexity and heterogeneity of the syndromes that are caused by mutations in NER genes suggests that the consequences of these mutations might go beyond the failure to remove DNA lesions. Intriguingly, the TFIIH factor has been initially identified as basal transcription factor functioning in the initiation phase of RNAPII-driven transcription (Egly and Coin, 2011). The helicase activity of TFIIH not only unwinds the double helix at sites of UV-induced lesions but also opens the promoter (**Figure 2**). In addition, TFIIH has been implicated in transcription that is mediated by RNAPI (Iben et al., 2002). Given the role in transcription, CS and TTD have been suggested to result primarily from transcription defects, rather than persistence of transcription-blocking lesions (Kamileri et al., 2012a). Indeed, not only TFIIH but evenly many other NER factors have recently been demonstrated to play a role in basal transcription (Le May et al., 2010). Furthermore, RNAPII stalling itself might have far-reaching consequences. For example CSB is not only required for initiating NER but also for re-initiating transcription upon UV irradiation (Proietti-De-Santis et al., 2006; **Figure 2**). Stalling of transcription complexes can also have detrimental consequences for the cell when for example a replication fork collides with a blocked RNAPII complex (Poveda et al., 2011).

Some of the degenerative phenotypes are likely to be caused by apoptotic responses to RNAPII stalling. During the repair process the NER complex is assembled in a highly dynamic manner as revealed by real-time imaging of green fluorescent protein (GFP)-tagged NER proteins in cell culture (Luijsterburg et al., 2010). This methodology is based on fluorescence recovery after photobleaching (FRAP) where subnuclear bleaching of the GFP tag allows visualization of the protein exchange with the unbleached fraction (Houtsmuller and Vermeulen, 2001). The dynamic FRAP experiments have been systematically performed for many NER factors. Intriguingly, it appears that the most time consuming step in the NER reaction is the verification of the damage (Luijsterburg et al., 2010). This verification step might be particularly important as CPDs form rather subtle alterations in the double helix structure and need to be distinguished from normal structural dynamics of chromatin.

The DNA binding properties of NER proteins have been probed by chromatin immunoprecipitation (ChIP) experiments (Le May et al., 2010). Here, the endogenous proteins are isolated together with the protein–DNA complex they are bound to. These studies revealed that NER proteins are bound at specific gene promoters and are needed for normal transcription independent of their DNA repair function (Le May et al., 2010; Kamileri et al., 2012b). Moreover, mutations in NER genes lead to a failure to initiate transcription of nuclear receptor-induced genes (Le May et al., 2010). Upon nuclear receptor activation the NER proteins XPC, CSB, XPA, XPG, and XPF–ERCC1 are recruited to the promoter. XPC, which previously was thought to function in GG-NER only, was found to initiate transcription, while CSB remains associated with RNAPII during elongation (**Figure 2**). XPC also mediates initiation of the transcription of the stem cell inducer *Nanog* together with Oct4/Sox2 (Fong et al., 2011). The XPF–ERCC1 and XPG endonucleases incise DNA proximal to the promoter and are required for the demethylation that precedes transcription initiation (Le May et al., 2012). Interestingly, *Ercc1* deficiency leads to cell differentiation failures. Gene expression analysis of *Ercc1* knockout mice showed strong resemblance with *Taf10* mutants (Kamileri et al., 2012b). TAF10 comprises a subunit of the TFIID complex that mediates transcription initiation. *Ercc1* like *Taf10* mutants fail to transcribe genes that are normally induced postnatally to facilitate developmental growth. Together, these data suggest that developmental abnormalities in NER mutants result from a failure to initiate developmental gene transcription programs. It remains to be established how transcription-blockage, re-initiation, and *de novo* initiation are interconnected. It seems conceivable that DNA damage surveillance in open reading frames might be tightly linked with transcription initiation and promoter clearance of RNAPII. In patients carrying mutations in NER, the requirement of NER factors for transcription might even exacerbate the consequences of the repair defect.

Not only factors acting in NER but also the proteins that are dysfunctional in HGPS and WS have been connected to transcription. Disruption of nuclear structure by transfection of a dominant negative lamin A mutant is followed by reduced transcription as measured by bromouridine-triphosphate (BrUTP) incorporation (Spann et al., 2002). Furthermore, the incorporation of nucleoside analogs and, therefore, efficient transcription has been reported to be impaired in cells with mutations in Werner’s helicase (Balajee et al., 1999). Consequently, it is likely that the highly complex progeroid syndromes result from imbalances in DNA metabolism, and in particular, transcription impediments that can be fueled by unrepaired DNA lesions.

## OUTLOOK: TARGETING TRANSCRIPTION IN THERAPY

Taken together several lines of evidence indicate that responses to transcription-blocking lesions are powerful tumor suppressors. Particularly, the apparent cancer protection in TC-NER defective patients suggests that cellular growth impairment together with pro-apoptotic signaling in response to RNAPII stalling could effectively limit cancer cell proliferation. Importantly, there are p53-dependent as well as -independent responses making transcription-blockage mediated tumor suppression also relevant when p53 is mutated (Ljungman and Lane, 2004; Garinis et al., 2009). Indeed, it was proposed that targeting of transcription might be a useful therapeutic strategy for cancer treatment (Ljungman and Lane, 2004). Several chemotherapies affect transcription and part of the effector mechanisms might actually depend on RNAPII stalling. More specifically, the illudin S derivative irofulven has been developed to target sarcomas (Paci et al., 2006). Illudin S and irofulven are highly cyotoxic to cells that are TC-NER deficient, indicating that the induced lesions lead to RNAPII blockage (Jaspers et al., 2002). Trabectedin (ET-743, Yondelis), in contrast, becomes toxic when the TC-NER reaction is activated and produces a cytotoxic repair intermediate (Takebayashi et al., 2001). The NER component ERCC1, through its functioning in ICL removal, has been implicated in the resistance to cisplatin, which is commonly used to treat lung carcinoma (McNeil and Melton, 2012). Moreover, cisplatin-induced DNA damage has been shown to be removed faster when NER proteins are overexpressed, whereas mutations in NER confer hypersensitivity to the treatment (Kelland, 2007). Given the involvement of NER, it has been suggested to apply fludarabine nucleoside in combination with cisplatin to treat cancer. Fludarabine could inhibit NER reaction at the repair synthesis step and by doing so, hypersensitize cells to lesions normally repaired by NER (Li et al., 1997). It will be important to establish whether status of NER factors can be predictive of therapy outcome and, likewise, whether targeting of NER might open new avenues to support specific types of chemotherapy. Also topoisomerase inhibitors that are frequently used in chemotherapy have been suggested to interfere with transcription. Camptothecin, inhibiting topoisomerase I, and doxorubicin, targeting DNA topoisomerase II, interfere with elongation and initiation of transcription, respectively, and are, therefore, efficient inducers of apoptosis in cancer cells (Ljungman and Lane, 2004).

The responses to transcription-blockage, namely attenuation of the somatotropic axis, might also provide targets for therapies aimed to prevent age-related diseases (Schumacher, 2009). An early shift to the maintenance program, for instance through inhibition of IGF-1R signaling, might delay the onset of age-related disease. *Igf-1r *mice are not only protected from aging but also from neurodegenerative diseases as demonstrated in mouse models for Alzheimer’s disease (Cohen et al., 2009; Freude et al., 2009). There are nearly a dozen compounds that target IGF-1 mediated signal transduction. These were originally developed for use in cancer treatment to antagonize IGF-1 mediated mitogenic signaling (Carter et al., 2002). Although many cancer cells are critically dependent on IGF-1 signaling, it is likely that due to their inherent genome instability cancer cells can mutate and bypass this requirement for continuous proliferation. Nonetheless, IGF-1 inhibitors might become useful in preventive treatment for age-related diseases. Consistent with this concept, inhibition of mTOR, a downstream component of IGF-1 signal transduction, by rapamycin extends lifespan of various mouse strains (Harrison et al., 2009).

In conclusion, it will be of pivotal importance to establish the role of transcription impairments in progeroid syndromes and during the course of DNA damage accumulation with aging. It will be particularly interesting to further explore the distinct consequences of impaired transcription elongation and initiation and to establish how cellular response mechanisms to stalled transcription complexes impact on the physiological adjustments of the aging organism.

## Conflict of Interest Statement

The authors declare that the research was conducted in the absence of any commercial or financial relationships that could be construed as a potential conflict of interest.
